# Direct evidence that the GPCR CysLTR2 mutant causative of uveal melanoma is constitutively active with highly biased signaling

**DOI:** 10.1074/jbc.RA120.015352

**Published:** 2020-12-11

**Authors:** Emilie Ceraudo, Mizuho Horioka, Jordan M. Mattheisen, Tyler D. Hitchman, Amanda R. Moore, Manija A. Kazmi, Ping Chi, Yu Chen, Thomas P. Sakmar, Thomas Huber

**Affiliations:** 1 Laboratory of Chemical Biology and Signal Transduction, The Rockefeller University, New York, New York, USA; 2 Tri-Institutional PhD Program in Chemical Biology, New York, New York, USA; 3 Human Oncology and Pathogenesis Program, Memorial Sloan Kettering Cancer Center, New York, New York, USA; 4 Louis V. Gerstner Jr. Graduate School of Biomedical Sciences, Memorial Sloan Kettering Cancer Center, New York, New York, USA; 5 Weill Cornell Graduate School of Medical Sciences, Cornell University, New York, New York, USA; 6 Department of Medicine, Memorial Sloan Kettering Cancer Center, New York, New York, USA; 7 Department of Medicine, Weill Cornell Medical College, New York, New York, USA; 8 Division of Neurogeriatrics, Department of Neurobiology, Care Sciences and Society, Karolinska Institutet, Solna, Sweden

**Keywords:** G protein–coupled receptor, G protein, β-arrestins, uveal melanoma, signaling, CysLTR2, biased signaling, constitutive activity, BRET, bioluminescent resonance energy transfer, BRET^2^, bioluminescent resonance energy transfer 2, BSA, bovine serum albumin, CA, constitutive activity, CAM, constitutively active mutant, CI, confidence interval, CysLTR2, cysteinyl-leukotriene receptor 2, DPBS, Dulbecco’s PBS, EPAC, exchange protein activated by cAMP, FBS, fetal bovine serum, GPCR, G protein–coupled receptor, HTRF, homogeneous time-resolved fluorescence immunoassay, IP_1_, d-myo-inositol-1-phosphate, LiCl, lithium chloride, LTD4, leukotriene D4, MV, missense variant, PLCβ, phospholipase C-β, RLuc3, *Renilla* Luciferase, UVM, uveal melanoma, V2(A)_6_, hexa-alanine variant

## Abstract

Uveal melanoma is the most common eye cancer in adults and is clinically and genetically distinct from skin cutaneous melanoma. In a subset of cases, the oncogenic driver is an activating mutation in *CYSLTR2*, the gene encoding the G protein–coupled receptor cysteinyl-leukotriene receptor 2 (CysLTR2). The mutant *CYSLTR2* encodes for the CysLTR2–L129Q receptor, with the substitution of Leu to Gln at position 129 (3.43). The ability of CysLTR2–L129Q to cause malignant transformation has been hypothesized to result from constitutive activity, but how the receptor could escape desensitization is unknown. Here, we characterize the functional properties of CysLTR2–L129Q. We show that CysLTR2–L129Q is a constitutively active mutant that strongly drives Gq/11 signaling pathways. However, CysLTR2–L129Q only poorly recruits β-arrestin. Using a modified Slack–Hall operational model, we quantified the constitutive activity for both pathways and conclude that CysLTR2–L129Q displays profound signaling bias for Gq/11 signaling pathways while escaping β-arrestin–mediated downregulation. *CYSLTR2* is the first known example of a G protein–coupled receptor driver oncogene that encodes a highly biased constitutively active mutant receptor. These results provide new insights into the mechanism of CysLTR2–L129Q oncoprotein signaling and suggest *CYSLTR2* as a promising potential therapeutic target in uveal melanoma.

The superfamily of G protein–coupled receptors (GPCRs) is the largest gene family encoding cell-signaling transmembrane proteins, and approximately one-quarter of ∼400 nonolfactory GPCRs are therapeutic drug targets. Large-scale genomic analysis has revealed that one in five individuals carries a missense variant (MV) in a clinically relevant GPCR gene. The rate of *de novo* germline MVs in a GPCR gene is one in every 300 newborns, and one in 7 MVs is observed at a potential functionally relevant site (1). In addition, GPCR genes are commonly mutated in cancer. Somatic mutations are found in 20% of tumor samples, but the lack of specific “hotspot” variants makes it difficult to identify and validate individual receptors as driver oncogenes (2).

We recently reported the discovery of a recurrent “hotspot” somatic missense mutation of the GPCR gene *CYSLTR2*. The mutant *CYSLTR2* encodes cysteinyl-leukotriene receptor 2 (CysLTR2)–L129Q that carries a single amino acid substitution at a highly conserved residue in helix 3 (Ballesteros–Weinstein generic position 3.43) (3) and serves as a driver oncogene in patients with uveal melanoma (UVM) (2). UVM is the most common intraocular malignancy and is associated with a high rate of metastasis with short survival time for patients (4). UVM shows a characteristic pattern of mutually exclusive activating mutations in the CysLTR2–Gq/11–PLCβ4 (phospholipase C-β4) pathway in almost all tumors (2, 5, 6). The same CysLTR2–L129Q mutation has also been identified as an oncogenic driver mutation in several other melanocytic tumors (7). CysLTR2 is a significantly mutated GPCR not only in UVM but also in gastrointestinal adenocarcinoma (8).

Here, we report that the UVM oncogene product CysLTR2–L129Q has a unique gain-of-function phenotype and report its precise signaling mechanism. We show that CysLTR2–L129Q is a constitutively active mutant (CAM) receptor that strongly couples to Gq/11 cellular signaling pathways. However, the receptor CAM only very weakly recruits β-arrestins and thereby avoids cellular downregulation mechanisms. We quantified the signaling bias of the mutant receptor by comparing the constitutive activity (CA) for the WT and L129Q mutant using values calculated from the modified Slack–Hall operational model. Finally, we showed that the receptor bias of the L129Q CAM toward Gq and away from β-arrestins is due to the unusual C-terminal sequence of the receptor.

## Results and discussion

### CysLTR2–L129Q signals through Gq/11-PLCβ pathways

To characterize the functional phenotype of CysLTR2–L129Q, we first determined the agonist-dependent signaling for CysLTR2–L129Q and CysLTR2 WT. CysLTR2 predominantly couples to Gq/11 when treated with the agonist leukotriene D4 (LTD4) (9). Phospholipase C-β (PLCβ) is the classical effector of Gq/11 and results in receptor-stimulated phosphoinositide hydrolysis that is conveniently monitored as an accumulation of d-myo-inositol-1-phosphate (IP_1_) in the presence of lithium chloride (LiCl) (10). We first obtained a time course of basal and LTD4-dependent IP_1_ accumulation in HEK293T cells transiently transfected with plasmids for CysLTR2 WT, CysLTR2–L129Q, and mock controls. LTD4-stimulated CysLTR2 WT showed increasing IP_1_ accumulation over the first 100 min before reaching a plateau (Fig. S1*A*), whereas the unstimulated CysLTR2 WT samples were indistinguishable from mock-transfected controls with and without LTD4 treatment. The samples transfected with the same amount of DNA encoding for the CysLTR2–L129Q mutant showed ligand-independent IP_1_ accumulation of comparable magnitude as LTD4-treated CysLTR2 WT. After 100 min, the basal IP_1_ accumulation of CysLTR2–L129Q kept increasing, whereas the ligand-dependent signaling of the WT receptor reached a plateau (Fig. S1*B*).

**Figure 1:**
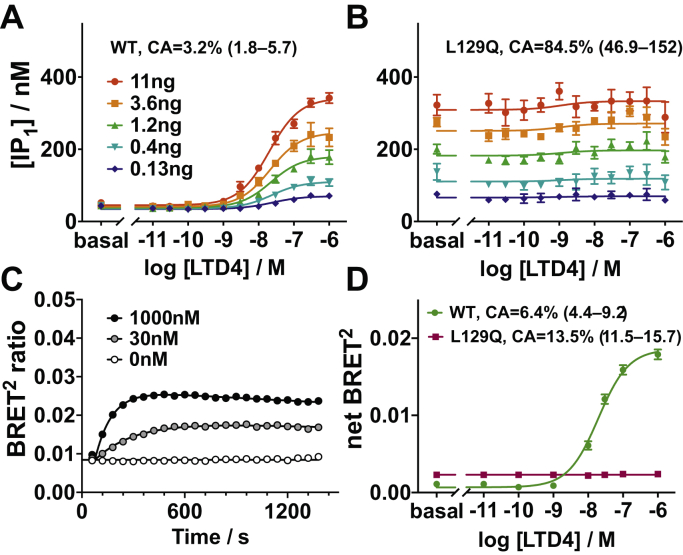
**Oncoprotein CysLTR2–L129Q is a Gq-biased CAM that only weakly recruits β-arrestin2**. (*A* and *B*) Gq second messenger IP_1_ accumulation assay. *A*, Agonist LTD4 leads to dose-dependent IP_1_ accumulation in HEK293T cells transfected with different amounts (0.13–11 ng DNA, *blue* to *red*) of CysLTR2-GFP10 WT. *B*, The corresponding experiment with CysLTR2–L129Q shows dramatic agonist-independent basal IP_1_ accumulation that scales with the amount of CysLTR2–L129Q–encoding DNA. Only a very small agonist-dependent response to LTD4 can be detected. The results show that CysLTR2–L129Q is a CAM with 85% constitutive activity relative to fully agonist-stimulated WT receptor. Data points are the mean ± SEM of the accumulated IP_1_ concentration and from one experiment with four replicates each. The set of curves are fits to the Slack–Hall operational model (Table S1*B*, Fig. 2). *C* and *D*, β-arrestin2-recruitment BRET^2^ assays. *C*, Time course of LTD4-stimulated β-arrestin2 recruitment for three LTD4 concentrations (0 nM, 30 nM, and 1000 nM). β-arrestin2 recruitment exhibits a biphasic time course, increasing for about 10 min after LTD4 addition before slowly decreasing. The data points are the mean ± SEM from three independent experiments with eight replicates each. Curves are double exponential fits (Table S1*C*). *D*, The LTD4 dose-dependent β-arrestin2 recruitment to CysLTR2 WT (*dark green*; EC_50_ is 30 nM (95% CI: 25–36 nM)). In comparison, CysLTR2–L129Q (*maroon*) shows higher basal β-arrestin2 recruitment, corresponding to 13.5% constitutive activity, and no response to agonist. The data points are the mean ± SEM from three independent experiments with three replicates each. CAM, constitutively active mutant; IP_1_, d-myo-inositol-1-phosphate; LTD4, leukotriene D4; BRET^2^, bioluminescence resonance energy transfer 2; CysLTR2, cysteinyl-leukotriene receptor 2.

**Figure 2:**
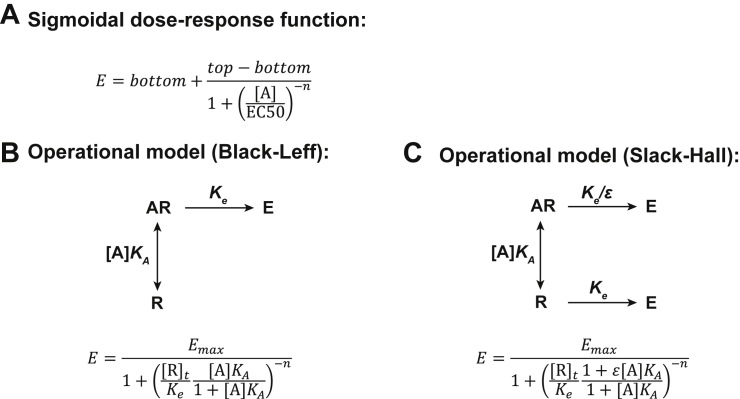
**Modeling the pharmacology of constitutively active receptors.** The different models and corresponding equations proposed to describe the pharmacology of the constitutively active CysLTR2-L129Q receptor are described here. *A*, the sigmoidal dose-response model, *B*, the Black–Leff operational model (11), and (*C*) the Slack–Hall operational model (12, 13). CysLTR2, cysteinyl-leukotriene receptor 2.

We generated fusion constructs of CysLTR2 WT and CysLTR2–L129Q, with a version of GFP (GFP10) at the C-terminus. These fusion constructs enable quantification of basal and agonist-dependent Gq/11 cellular signaling and β-arrestin recruitment activity under comparable conditions. The agonist LTD4 induces a dose-dependent increase in IP_1_ accumulation for CysLTR2 WT (Fig. 1*A*) that scales as expected with the receptor density controlled by the gene dosage as described by a modified Slack–Hall operational model (Fig. 2, Table S1*B*). In contrast, the CysLTR2–L129Q mutant shows little or no response to treatment with LTD4, but the ligand-independent basal IP_1_ accumulation dramatically increases with increasing gene dosage (Fig. 1*B*). Therefore, the mutation is meeting the essential criteria for a CAM because the hallmark of CAM receptors is agonist-independent signaling that scales with receptor density. The results also show that under the experimental conditions, the Gq signaling pathway is not saturated, which suggests that the high basal receptor activation of Gq for CysLTR2–L129Q is not due to a high amplification of the Gq signaling pathway.

### Characterizing the β-arrestin recruitment to CysLTR2–L129Q

Signals from active GPCRs are normally terminated by β-arrestin–dependent mechanisms, including desensitization, sequestration, and downregulation. We next asked the question, how is CysLTR2–L129Q capable of sustained strong signaling at a level comparable with the fully agonist-stimulated WT receptor? CysLTR2 has been shown to bind β-arrestin2 in response to several agonists (14). However, little is known about the β-arrestin–dependent desensitization, trafficking, and downregulation of CysLTR2 and CysLTR2–L129Q.

We designed a bioluminescence resonance energy transfer 2 (BRET^2^) experiment to quantify the basal and agonist-dependent binding of β-arrestins to CysLTR2 variants. The BRET^2^ experiments are performed on HEK293T cells expressing CysLTR2–GFP10 fusion construct in combination with β-arrestins fused to an engineered variant of *Renilla* luciferase (RLuc3), β-arrestin–RLuc3 (15). We performed a time-course experiment to characterize the agonist-dependent β-arrestin recruitment. The BRET^2^ ratio shows an agonist concentration–dependent increase for approximately 10 min after the addition of the agonist LTD4, before starting to decrease again slowly (Fig. 1*C*). The initial increase in the slope increases with higher concentrations of the agonist. The shapes of the time courses were similar when comparing samples expressing β-arrestin1–RLuc3 and β-arrestin2–RLuc3, but the peak increase seen for β-arrestin2–RLuc3 was almost twice that of β-arrestin1–RLuc3 (Fig. S2). Such a biphasic BRET^2^ β-arrestin recruitment time course is typical for GPCRs with “class A” β-arrestin–recruitment phenotype that have transient, weak interactions with β-arrestins. These class-A receptors rapidly recycle after internalization (16).

The LTD4 dose-dependent increase of the BRET^2^ ratio for samples transfected with CysLTR2–GFP10 WT and β-arrestin–RLuc3 substantiate the finding from the time-course assay that the agonist-dependent increase of BRET^2^ is larger for β-arrestin2–RLuc3 as compared with β-arrestin1–RLuc3 (Fig. S3*A*). Although the agonist-dependent increase was different, the midpoints of the sigmoidal fits of the agonist dose-dependent data for both β-arrestins were identical (Fig. S3*B*). From these findings, we decided to proceed with the subsequent experiments using only β-arrestin2, which gave the larger BRET^2^ ratios.

### CysLTR2–L129Q poorly recruits β-arrestins

To characterize the effect of the L129Q mutation on β-arrestin recruitment, we included a set of samples expressing CysLTR2–L129Q in the BRET^2^ experiments. The results from the LTD4 dose-response experiment show a basal, ligand-independent net BRET^2^ ratio of 0.0028 ± 0.00007, with no ligand dose dependence (Fig. 1*D*). In comparison, the basal net BRET^2^ ratio for the WT receptor is 0.0007 ± 0.0002 that increases to 0.0186 ± 0.0004 at saturating LTD4 concentrations (Table S1*A*).

### CysLTR2–L129Q is a Gq-biased CAM that escapes β-arrestin–mediated downregulation

We next quantified the CA for both Gq/11 signaling and β-arrestin recruitment using the modified Slack–Hall operational model to enable the calculation of *receptor bias* between Gq/11 and β-arrestin pathways (12) for L129Q relative to WT. The term *receptor bias* was introduced to describe the pathway preference of the basal signaling activity of a receptor (12), in contrast to the term *agonist bias* that describes ligand-dependent pathway preferences of a receptor (17).

Figure 2 introduces two operational models: the Black–Leff and Slack–Hall models. The key insight of Zhou *et al.* is that the Slack–Hall model can be used to quantify the inherent agonist-independent pathway bias of the constitutive signaling of a receptor referred to as *receptor bias* (12, 13). The Slack–Hall model is an expansion of the classical Black–Leff operational model, which underlies methods to calculate functional selectivity or *agonist bias* (17). In the Slack–Hall model, both the free receptor [R] and agonist-bound receptor [AR] can produce a stimulus, S=ε[AR]+[R]. The parameter *ε* describes the efficacy of an agonist (A), to produce a stimulus. The Slack–Hall model splits the τ parameter of the Black-Leff model into a product of two parameters, χ and ε. The basal response is determined by χ and is defined as the ratio of [R]_*t*_, the total receptor concentration, and *K*_*e*_, the receptor concentration producing half-maximal effect in the *absence* of an agonist. In contrast, the τ parameter in the Black-Leff model is the ratio of [R]_*t*_ and a different *K*_*e*_, which is defined as the receptor concentration producing half-maximal effect in the *presence* of a saturating agonist concentration. The ε parameter measures the *intrinsic efficacy* of the ligand. We slightly modified the original form of this equation to account for fitting problems for χ, the basal response parameter, in cases where the CA is very low. Taking the *log τ* parameter from the Black-Leff model, we implicitly calculate *log χ* from *log τ*–*log ε* (refer to Experimental procedures).

**Figure 3:**
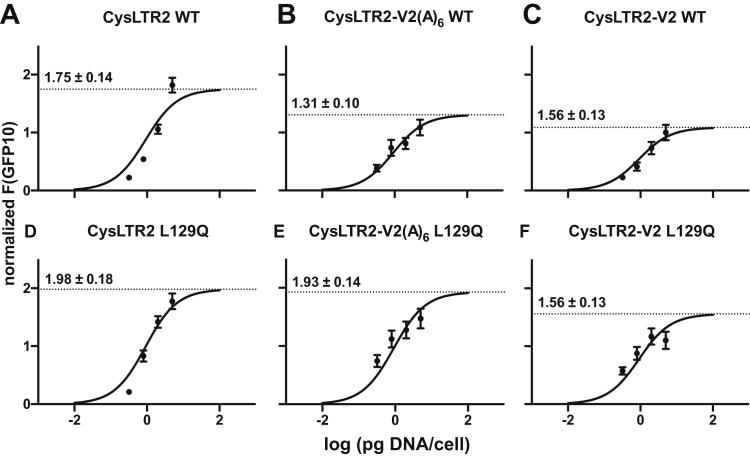
**Gene dosage-dependent receptor expression.** Normalized GFP10 fluorescence (F(GFP10)) of HEK293T cells transfected with varying amounts of receptor-encoding plasmid are fit to a sigmoidal model, where F(GFP10) is the output as a function of DNA dosage. 40,000 cells were transfected with 12.5, 32, 80, and 200 ng of receptor-encoding plasmid, to yield 0.3125, 0.8, 2, and 5 pg/cell of DNA. The log EC_50_ value (log EC_50_ = −0.038 ± 0.072) is shared for all CysLTR2 WT (*A*, normal; *B*, V2(A)_6_ tail; *C*, V2 tail) and L129Q (*D*, normal; *E*, V2(A)_6_ tail; *F*, V2 tail) variants. The bottom parameter is set to zero, whereas the top parameter is left free to capture the different expression levels of each variant and is indicated on the graphs. These sigmoidal fits are used to interpolate F(GFP10) for subsequent plots (Fig. 4) because the F(GFP10) is not strong enough to reliably measure the total receptor concentrations, especially at lower DNA/cell levels used in the Gq second messenger IP_1_ assays. IP_1_, d-myo-inositol-1-phosphate; V2(A)_6_, hexa-alanine variant; CysLTR2, cysteinyl-leukotriene receptor 2.

The parameter χ determines the value of the basal response. The challenge is that χ is proportional to the receptor density, which requires standardization for the comparison of receptor mutants with potential impact on receptor expression levels. In our experiments, we control the receptor density by the gene dosage and measure the fluorescence from the GFP10 fusion to calibrate the relative expression levels (Fig. 3). The GFP10 readings were normalized and fit to a sigmoidal model, where GFP10 fluorescence, designated as F(GFP10), is the response as a function of DNA dosage.

**Figure 4:**
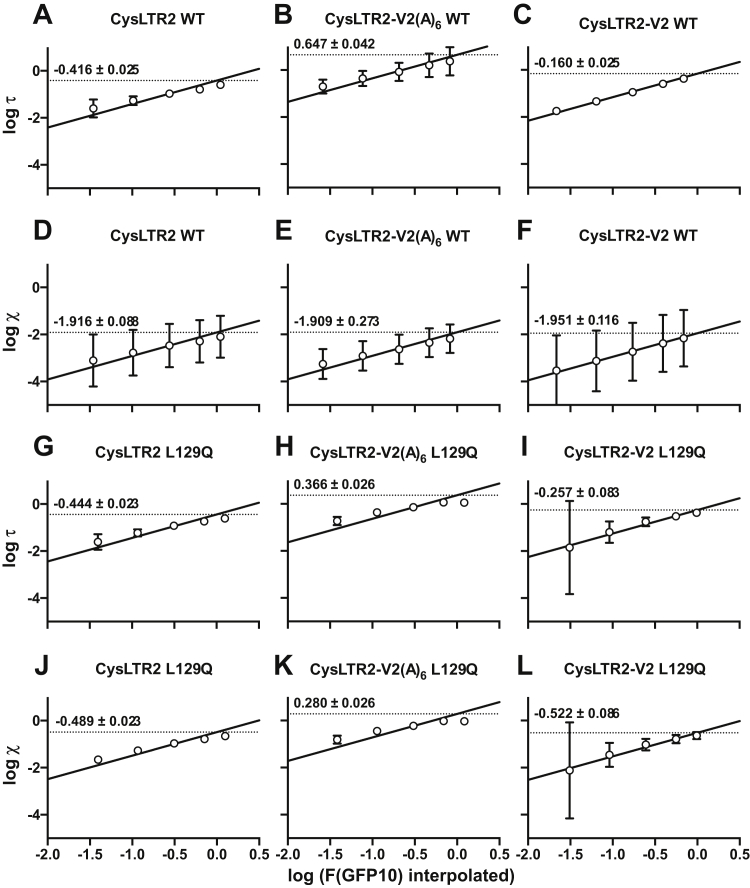
**Basal and agonist-dependent receptor activity for Gq pathway as a function of receptor expression.** The F(GFP10) readings for HEK293T cells transfected with varying amounts of receptor-encoding plasmid from the Gq second messenger IP_1_ assays (Figs. 1, *A*–*B*, 6, *A*–*D*) were interpolated from the sigmoidal fits in Figure 3. 7000 cells were transfected with 0.1, 0.4, 1.2, 3.6, and 11 ng of receptor-encoding plasmid, to yield 0.019, 0.057, 0.17, 0.51, and 1.57 pg/cell of DNA. These were plotted against *log τ* values (WT, *A–C*; L129Q, *G–I*) and *log χ* values (WT, *D–F*; L129Q, *J–L*) obtained from fitting the data to the modified Slack–Hall model (Table S1*B*). Assuming the F(GFP10) is proportional to the total receptor concentration by some scaling constant, *c*, and rearranging $\chi =\frac{{\left[R\right]}_{T}}{K_{e}}$ gives $log\chi =logc+\log\left(F\left(GFP10\right)\right)-logK_{e}$. Plotting *log χ* against *log (F(GFP10))* and fitting to a line with a slope of 1 gives y-intercept of *log c* – *log K*_e_. We can similarly plot *log τ* against *log (F(GFP10))* to get a y-intercept of *log c* – *log K*_e_ + *log ε*. The *y*_*0*_ values are indicated on graphs and allow for an accurate quantification of *log K*_e_ and *log ε*, of different receptor constructs at standard density. IP_1_, D-myo-inositol-1-phosphate.

We used the sigmoidal fits of the F(GFP10) to adjust for lower DNA/cell levels used in the Gq second messenger IP_1_ assays, and thus the interpolated F(GFP10) values were plotted against *log τ* values and *log χ* values from fitting the data to the modified Slack–Hall model (Fig. 4, Table S1*B*). Assuming the F(GFP10) is proportional to the total receptor concentration by some scaling constant, *c*, and rearranging $\chi =\frac{{\left[R\right]}_{T}}{K_{e}}$ gives the following:

$$log\chi \;=\;logc\;+\;\log\left(F\left(GFP10\right)\right)\;-\;logK_{e}$$

Thus, plotting *log χ* against *log (F(GFP10))* and fitting to a line with a slope of 1 gives y-intercept of *log c* – *log K*_e_. We can similarly plot *log τ* against *log (F(GFP10))* to get a y-intercept of *log c* – *log K*_e_ + *log ε*. These allow for an accurate quantification of *log ε* and of differences of *log K*_e_ for different receptor constructs at a standard density.

**Figure 5:**
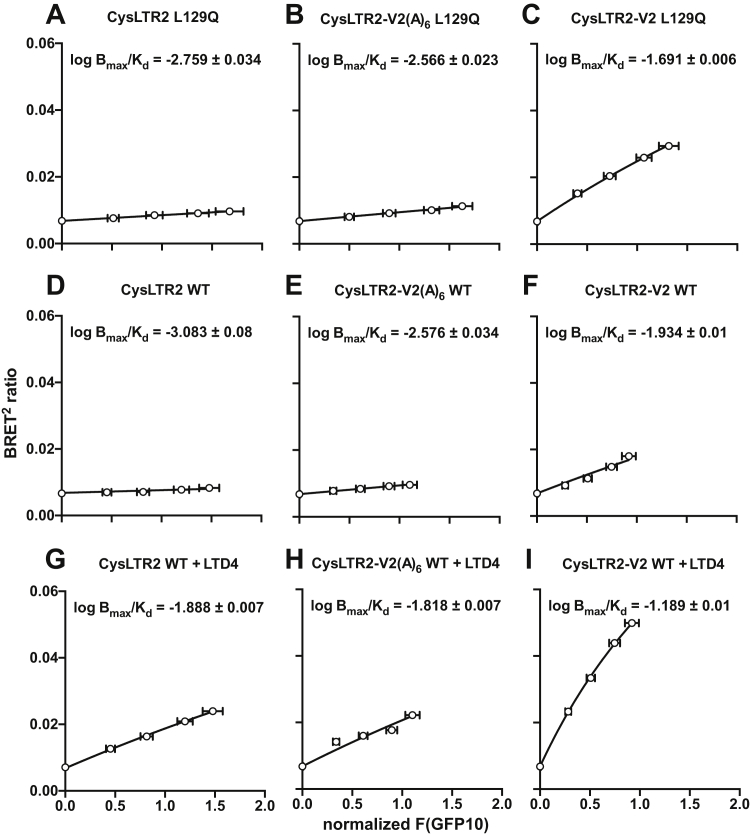
**Basal and agonist-dependent receptor activity for β-Arrestin2 pathway as a function of receptor expression.** Saturation-binding BRET^2^ experiment with CysLTR2–L129Q (*A–C*), unstimulated WT (*D–F*) and WT stimulated with 1000-nM LTD4 (*G–I*). The data are fit to a one-site saturation binding function. Tight independent estimates of *K*_*d*_ and *B*_*max*_ are not required because at low concentrations, only the ratio *B*_*max*_:*K*_*d*_ determines the concentration-dependent binding, which can be estimated from the initial slope. The initial slopes are well defined by samples even at low expression levels of receptors and avoid the need for very high receptor concentrations to reach saturation. All data are fit with a shared *log B*_*max*_, and *log B*_*max*_/*K*_*d*_ are indicated on the graphs. This enables direct comparison of the slopes for unstimulated CysLTR2 WT and CysLTR2–L129Q to subsequently calculate the constitutive activity values in Figures 1*D* and 6, *E–F*. BRET^2^, bioluminescence resonance energy transfer 2; LTD4, leukotriene D4; CysLTR2, cysteinyl-leukotriene receptor 2.

We noticed that in the absence of a ligand, the Slack–Hall model reduces to the mathematical form of a one-site saturation-binding function (18). We plotted the BRET^2^ ratios against normalized F(GFP10) readings for each CysLTR2 variant and fit the data to a one-site saturation-binding isotherm accordingly (Fig. 5).

Tight independent estimates of *K*_*d*_ and *B*_*max*_ are not required because at low concentrations, only the ratio *B*_*max*_:*K*_*d*_ determines the concentration-dependent binding, which can be estimated from the initial slope. The initial slopes are well defined by samples even at low expression levels of receptors and avoid the need for very high receptor concentrations to reach saturation. The initial slope of a saturation binding experiment as a function of the total receptor concentration is *B*_*max*_*/K*_*d*_. The initial slope of the Slack–Hall model as a function of the total receptor concentration is *E*_*max*_*/K*_*e*_. The equivalence of *B*_*max*_*/K*_*d*_ and *E*_*max*_*/K*_*e*_ enables subsequent calculations of Δlog χ, the differences of log χ for the WT and mutant receptors that determine changes in the CA, and the double difference ΔΔlog χ that determines the receptor bias. Those differences eliminate the constant *B*_*max*_ and *E*_*max*_ terms and focus on changes of the bias-relevant terms *K*_*d*_ and *K*_*e*_.

Using the values obtained from the fits in Figures 4 and 5, we are finally able to compare CAs for the different receptor constructs at a standard density. The CAs are normalized relative to the fully agonist-stimulated WT receptor. The ligand-independent CA of CysLTR2–L129Q corresponds to 84.5% (95% confidence interval [CI]: 46.9%–152%) of the maximally LTD4-stimulated CysLTR2 WT at a comparable total receptor concentration. The CA of the WT receptor is 3.2% (95% CI: 1.8%–5.7%). Therefore, the L129Q mutation results in a 26-fold increase of the CA in the Gq pathway. The ligand-dependent increase in IP_1_ signaling of CysLTR2–L129Q is statistically insignificant as reflected by the efficacy parameter ε of 1.11 (95% CI: 1.03–1.19) close to unity (Table S1*B*). Overall, the results suggest that CysLTR2–L129Q displays a gain-of-function phenotype for ligand-independent basal signaling and a loss-of-function phenotype for agonist-dependent signaling in the Gq/11 signaling pathway. Compared with the agonist-dependent β-arrestin recruitment of the WT receptor (set to 100%), the CA of the WT receptor is 6.4% and that of the L129Q mutant is 13.5%. Therefore, the effect of the L129Q mutation on the CA in the β-arrestin pathway is a 2-fold increase, which is much smaller than the 26-fold increase in the Gq pathway. Therefore, the L129Q mutation introduces a strong bias of the constitutive signaling (“receptor bias”) toward Gq and away from β-arrestins.

It is challenging to quantify receptor bias. Although ligand bias or biased agonism has been studied for many GPCR–agonist pairs with well-developed mathematical approaches, the concept of receptor bias is still a relatively underexplored area. To our knowledge, this is the first time a rigorous mathematical method has been developed for the detailed analysis of receptor bias. The method described here is generalizable and can be used to study mutations that might cause receptor bias in any GPCR.

### Enhanced recruitment of β-arrestin has only a small effect on basal Gq activation in CysLTR2–L129Q

We speculated that the receptor bias away from β-arrestins may be due to a lack of phosphorylation sites in the C-terminal tail of the receptor. The CysLTR2 sequence SVWLRKE has been predicted to be a partial phosphorylation code characteristic of a “class A” β-arrestin–recruitment phenotype (19), with transient and weak interactions with β-arrestins, consistent with our findings of a biphasic β-arrestin–recruitment time course. We hypothesized that adding a sequence of the vasopressin V2 receptor, which carries a strong phosphorylation code, would enhance the recruitment of β-arrestins and switch the receptor to a “class B” β-arrestin–recruitment phenotype. Class B recruitment phenotype corresponds to tight and more stable binding of β-arrestins, which leads to the GPCR-β–arrestin complex being sustained for a longer time (16). We used a hexa-alanine variant (V2(A)_6_) as a negative control in which we replaced six Ser and Thr residues with Ala residues (20). To explore the strong bias of CysLTR2–L129Q toward Gq, we must investigate the relationship between β-arrestin and Gq binding to CysLTR2. To this end, we enhanced the β-arrestin recruitment to CysLTR2 through the addition of a strong phosphorylation code and observed the corresponding effects on Gq binding and activation. By enhancing the β-arrestin binding to CysLTR2–L129Q, is it possible to observe a shift in the bias?

**Figure 6:**
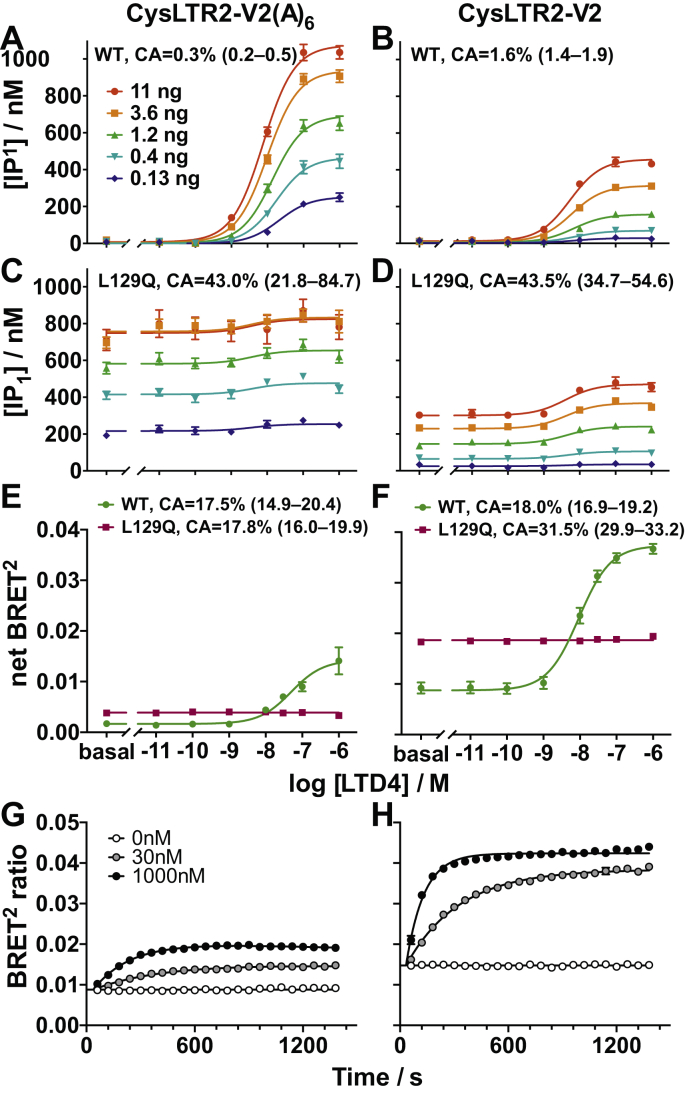
**Recruitment of β-arrestin has only small effect on basal Gq activation in CysLTR2–L129Q.** We added the C-terminal 27 residues of the vasopressin V2 receptor to full-length CysLTR2 to promote high-affinity interactions with β-arrestins (construct CysLTR2-V2). Construct CysLTR2–V2(A)_6_ is the corresponding phosphorylation-resistant control. *A–D*, Gq second messenger IP_1_ accumulation assay. *E–H*, β-arrestin2–recruitment BRET^2^ assay. *A* and *B*, Agonist-stimulated Gq signaling is reduced by the addition of the V2 sequence as compared with the addition of V2(A)_6_ sequences at comparable gene dosages. *C* and *D*, Similarly, basal Gq signaling of L129Q is reduced by half in CysLTR2-V2 as compared with -V2(A)_6_. Data points are the mean ± SEM from three independent experiments with six replicates each. Sets of curves are fits to the Slack–Hall operational model (Table S1*B*). *E* and *F*, Agonist-stimulated β-arrestin recruitment is enhanced by the V2 sequence and reduced by the V2(A)_6_ sequence. Basal, agonist-independent β-arrestin recruitment of L129Q is 5-fold stronger for CysLTR2-V2 than for -V2(A)_6_. Data points are the mean ± SEM from three independent experiments with three replicates each. *G* and *H*, Time course of LTD4-stimulated β-arrestin2 recruitment for three LTD4 concentrations (0 nM, *white circles*; 30 nM, *gray circles*; 1000 nM, *black circles*). While the CysLTR2-V2(A)_6_ WT exhibited a biphasic time course, CysLTR2-V2 WT reached a plateau after about 8 min. Data are fit to double-exponential curves (Table S1*C*), and points are the mean ± SEM from three independent experiments with eight replicates. LTD4, leukotriene D4; V2(A)_6_, hexa-alanine variant; CysLTR2, cysteinyl-leukotriene receptor 2.

The results from IP_1_ and BRET^2^ assays of the V2 and V2(A)_6_ tail variants for CysLTR2 WT and L129Q are shown in Figure 6. The V2 tail reduces the agonist-dependent signaling at comparable gene dosage for WT as compared with the V2(A)_6_ tail variant (Fig. 6, *A*–*B*). Similarly, the basal signaling of the L129Q mutant is reduced for V2 as compared with V2(A)_6_ (Fig. 6, *C*–*D*). Once more, Figure 3, Figure 4, Figure 5 were used to compare the CAs for the different receptor constructs at a standard density. The CA values of the L129Q mutants are comparable with 43.5% for the -V2 tail and 43.0% for the V2(A)_6_ tail variant. Surprisingly, the V2 tail restores some of the agonist-dependent signaling of L129Q as indicated by an efficacy parameter ε of 1.84 (95% CI: 1.70–1.99) different from unity (Table S1*B*). The V2 tail enhances both basal and agonist-dependent increase of BRET^2^ for CysLTR2 WT (Fig. 6, *E*–*F*). Interestingly, the enhancement of basal, agonist-independent β-arrestin recruitment to CysLTR2–L129Q was much more pronounced for the V2 tail variant than with the V2(A)_6_ control, which showed only weak β-arrestin recruitment similar to that of CysLTR2–L129Q without the added sequences. We also determined the agonist-dependent β-arrestin–recruitment time course for CysLTR2 WT that showed a biphasic time course for the V2(A)_6_ control and a monophasic increase for the V2 construct, which is consistent with a “class B” β-arrestin–recruitment phenotype and with tightly bound β-arrestin (Fig. 6, *G*–*H*). The CA significantly increases from 18.0% for WT to 31.5% for L129Q in the -V2 tail variant, whereas the V2(A)_6_ variants have comparable CA values or 17.5% (WT) and 17.8% (L129Q). Therefore, the V2 tail reduces the receptor bias of L129Q away from β-arrestins. We conclude that the receptor bias of the L129Q CAM toward Gq and away from β-arrestins is due to the C-terminal sequence of the receptor.

Why does the enhanced β-arrestin recruitment not interfere more strongly with the Gq-activation–dependent IP_1_ accumulation? The binding of β-arrestins with an activated receptor includes two interaction modes, one mode that preactivates β-arrestin, with interactions only with the phosphorylated tail of the receptor and a second fully activated mode, with simultaneous interactions with the core and the phosphorylated tail of the receptor blocking G protein signaling (21). The recent structure of a GPCR–G protein–β-arrestin megacomplex demonstrates how one receptor can simultaneously interact with a G protein, bound to the transmembrane core, and with β-arrestin, bound to the tail, without blocking G protein signaling (22). In contrast to the G protein, the structural determinants of the β-arrestin interactions with the receptor core are largely unknown because only the complex with rhodopsin has been solved so far (21, 23).

Based upon the available structural and biochemical data, we conclude that with CysLTR2–L129Q, the β-arrestin is unable to compete strongly with the G protein for the core binding site. Even by adding the V2 tail sequence to the mutant, where the β-arrestin is forced to interact strongly with the phosphorylated tail of the receptor, the β-arrestin is unable to simultaneously interact with the core to enter the fully activated mode. This is why a marked decrease in Gq activity is not observed. It is possible that this weak competition with the G protein is due to a receptor core conformation that is incompatible with β-arrestin binding but suitable, and even favorable, for G protein binding and activation. We speculate that the strong receptor bias of CysLTR2–L129Q toward Gq signaling is due to a selective stabilization of an intermediate state that is partially activated, perhaps facilitated by the L129Q mutation. In the intermediate state, G protein binding is promoted by the open pocket at the core. Catalytic activation of the nucleotide exchange in the G protein only transiently requires a fully active receptor state, whereas the stable interaction of β-arrestins with the receptor core requires a fully active state, consistent with our findings. We plan to investigate further the mechanisms behind the receptor bias in CysLTR2–L129Q.

In conclusion, we characterized a *CYSLTR2* oncogene in UVM and established that CysLTR2–L129Q drives Gq/11 signaling activity in malignant UVM and serves as a driver oncogene. We established that the CysLTR2–L129Q CAM is highly biased toward Gq/11 cellular signaling pathways and fails to recruit significantly β-arrestins. The lack of a strong phosphorylation code in the cytoplasmic tail contributes to the signaling bias of the extremely high CA of the mutant receptor. The biased constitutive signaling pattern of CysLTR2–L129Q explains why it can persistently activate Gq and avoid β-arrestin–dependent cellular downregulation mechanisms.

## Experimental procedures

We generated a CysLTR2–GFP10 fusion construct that enables quantification of basal and agonist-dependent Gq/11 cellular signaling and β-arrestin recruitment activity, as well as total receptor expression, under comparable conditions. Experiments were conducted on HEK293T cells transfected with the plasmids encoding the fusion constructs. The downstream Gq/11 activity was quantified using the CisBio IP-One homogeneous time-resolved fluorescence immunoassay (HTRF). The β-arrestin–recruitment activity was quantified using a BRET^2^ assay.

### Materials

LTD4 was from Cayman Chemical (Ann Arbor, MI). YM-254890 was from Wako Pure Chemical Industries (Richmond, VA). BRET substrate methoxy e-Coelenterazine was from NanoLight Technology (Pinetop, AZ). The IP-One HTRF kit was from CisBio (Codolet, France). Bovine serum albumin (BSA) fraction V, fatty acid-free, was from Roche (Basel, Switzerland). Poly-d-lysine and LiCl were from Sigma-Aldrich (St Louis, MO). HEK293T cells were from the American Type Culture Collection (Manassas, VA). Dulbecco’s modified Eagle’s medium (DMEM) GlutaMAX, FluoroBrite DMEM, Dulbecco’s PBS (DPBS) without calcium and magnesium, and HEPES buffer were from Thermo Fisher Scientific (Waltham, MA). Penicillin/Streptomycin (10,000 U/ml), L-glutamine, and Lipofectamine 2000 were from Thermo Fisher Scientific (Waltham, MA). Fetal bovine serum (FBS) was from Gemini Bio-Products (West Sacramento, CA). Black and white low-volume 384-well microplates, and black CELLSTAR 96-well microplates (polystyrene wells, flat bottom) were from Greiner (Monroe, NC). NEBuilder Hifi DNA Assembler, Dpn1, T4 DNA Ligase, Q5 Hot Start High-Fidelity DNA Polymerase, and dNTPs were from New England BioLabs (Ipswich, MA). QuikChange Lightning Site-Directed Mutagenesis Kit was from Agilent Technologies (Santa Clara, CA) and TagMaster Site-Directed Mutagenesis Kit was from GM Biosciences Inc (Frederick, MD). Oligonucleotides which are listed in Table S2 were purchased at the standard desalting grade from Integrated DNA Technologies (Coralville, IA). QIAGEN Plasmid Maxi Kits and QIAprep Spin Miniprep Kit were from QIAGEN (Germantown, MD).

### Molecular biology

#### CysLTR2–1D4 expression constructs

The synthetic vector encodes human CysLTR2 cDNA in pcDNA3.1(+) fused to an N-terminal FLAG tag (DYKDDDDK) and a C-terminal 1D4 epitope tag (TETSQVAPA) (2). The FLAG tag was then deleted by site-directed mutagenesis using the TagMaster Site-Directed Mutagenesis Kit according to the manufacturer’s instructions to generate CysLTR2-1D4. TagMaster primers that were used to generate the 1D4 constructs are listed in Table S2*A* and were purchased from Integrated DNA Technologies. All constructs were confirmed by sequencing (Genewiz, South Plainfield, NJ).

#### CysLTR2–GFP10 fusion protein construct

##### Primer design

The NEBuilder HiFi DNA Assembly Tool was used to assemble the BRET^2^ acceptor constructs CysLTR2–GFP10. These were assembled from three parts: pcDNA3.1(+) backbone from construct HA-CLIP-CLR (24), CysLTR2, and full-length C-terminal 1D4 epitope tag from FLAG-CysLTR2-1D4 mentioned above, and GFP10 from YB124_CXCR4–GFP10 (15). The primers (Table S2*B*) were designed using the NEBuilder Assembly Tool on the NEB website with a specific sequence to prime to the gene of interest for template priming (3’ end), as well as an overlap sequence to aid in assembly (5’ end).

##### PCR amplification of fragments

The fragments introduced above were PCR-amplified using Q5 Hot Start High-Fidelity DNA Polymerase and fresh dNTPs purchased from NEB. Briefly, the PCRs were performed in 25-μl total volume containing: 1× Q5 reaction buffer, 0.2-mM dNTPs, 0.5 μM of the forward primer, 0.5 μM of the reverse primer, 1-ng template DNA, and 1 unit of Q5 Hot Start High-Fidelity DNA Polymerase. The PCR thermocycle was as follows: initial denaturation at 98 °C for 30 s, followed by 25 cycles of denaturation (98 °C, 10s), annealing (varied from 50–70 °C, 30 s), and elongation (72 °C, 3 min), and ending with a final elongation (72 °C, 2 min). The recommended annealing temperature calculated on the NEBuilder Assembly Tool was used for each primer pair. After PCR, 1 unit of DpnI was added and the mixture was incubated at 37 °C for 30 min to digest any remaining template DNA. This was cleaned up, and any enzymes were removed using a DNA Clean and Concentrator (Zymo Research). The concentrations of all PCR-amplified fragments were determined using a NanoDrop.

##### Isothermal assembly

The NEBuilder Hifi DNA Assembler includes three enzymes: the exonuclease to create 3’ overhangs to aid annealing of neighboring fragments sharing a complimentary overlap region, the polymerase to fill the gaps of each annealed fragment, and the DNA ligase to seal nicks in the assembled DNA. The assembly reaction was performed in 20-μl total volume, with 50 ng of the vector, 100 ng of insert(s), and 10 μl of the NEBuilder HiFi DNA Assembly Master Mix. This was incubated at 50 °C for 60 min, and 2 μl of the assembled product was used to transform NEB 5-alpha competent *Escherichia coli* cells. Cells were spread on LB-Amp plates, and colonies were picked and confirmed by sequencing.

After the assembled product was confirmed by sequencing, the second *NotI* site that is flanked by two *XhoI* sites, which had been part of the pcDNA3.1(+) backbone in HA-CLR-CLIP, was removed. This was simply performed by digesting at the *XhoI* sites and self-ligating the vector using T4 DNA Ligase. We then sequenced the *NotI*-removed CysLTR2–GFP10 in its entirety to check for any erroneous modifications or linkages.

The β-arrestin2-RLuc3 BRET^2^ donor was constructed previously by fusing the coding sequence of RLuc3 to the C-terminus of β-arrestin2 (15).

#### CysLTR2-V2 and CysLTR2-V2(A)_6_ constructs

The cDNA encoding the 27 amino acids from the C-terminal tail of the vasopressin V2 receptor, GRTPPSLGPQDESCTTASSSLAKDTSS, was fused to the end of the full-length CysLTR2 receptor in the CysLTR2–GFP10 construct introduced above. As a negative control, we also fused a hexa-Ala variant (20) of the 27 amino acids with the phosphorylation sites (Ser and Thr) replaced with Ala (GRTPPSLGPQDESCTTAAAALAKDAAA, Ala substitutions underlined) to the full-length CysLTR2 receptor. For both of these amino acid sequences, we used the GeneOptimizer algorithm in GeneArt (Thermo Fisher Scientific, Waltham, MA) to get a DNA sequence optimized for humans and avoiding major restriction sites. The NEBuilder HiFi DNA Assembly Tool was used to assemble these constructs from two parts: the single-stranded oligonucleotide of the V2 receptor tail sequence and the linearized CysLTR2–GFP10 construct cut after the full-length receptor and before the GFP10. The primers were designed using the NEBuilder Assembly Tool on the NEB website, and their sequences are shown in Table S2*C*. The PCR amplification of the CysLTR2–GFP10 fragment and the isothermal assembly of these two parts were performed as outlined above. Note that the NEBuilder HiFi DNA Assembly method allows the assembly of a single-stranded oligonucleotide to a double-stranded DNA strand. All constructs were prepared from the QIAGEN Plasmid Maxi Kits and confirmed by Sanger sequencing using standard BGH reverse-sequencing primers.

### Signaling assays

#### IP_1_ accumulation assay

HEK293T cells were maintained in DMEM GlutaMAX supplemented with 10% FBS (passage numbers 5–14) at 37 °C under 5% CO_2_. Cells were transiently transfected directly ‘in-plate’ in low-volume 384-well plates using Lipofectamine 2000 according to manufacturer’s instructions with some modifications. The total DNA amount was kept constant at 11 ng per well using empty vector pcDNA3.1(+). All transfection reagents mixes were performed in DMEM GlutaMAX, unless specifically noted as being performed in FluoroBrite DMEM (DMEM without phenol red, described later). Briefly, the appropriate amount of plasmid DNA was mixed with DMEM (no FBS). In a separate mixture, the total Lipofectamine 2000 (2.5 μl per μg DNA) was mixed in DMEM (no FBS) and incubated for 5 min. The appropriate amount of Lipofectamine 2000/DMEM mixture was mixed with the DNA/DMEM and incubated for 20 min. Cells were then trypsinized, resuspended in DMEM supplemented with 20% FBS, and counted. Cells were mixed with the DNA/Lipofectamine 2000/DMEM mixture and directly plated onto 0.01% poly-d-lysine–coated, white, clear-bottom, tissue culture–treated low-volume 384-well plates at a density of 7000 cells per well in 7 μl. For the DNA titration assay with the CysLTR2-V2 tail variants, cells were similarly transfected in FluoroBrite DMEM and plated in 0.01% poly-d-lysine–coated, black, clear-bottom, tissue culture–treated low volume 384-well plates. All assays were conducted 24 h after the transfection. All plasmids were prepared from QIAGEN Plasmid Maxi Kits, resulting in a high-quality and high-concentration plasmid solution (about 1000–4000 ng/μl) unless otherwise specified. For specific experiments, the plasmids were prepared from the QIAprep Spin Miniprep Kit resulting in 10-fold lower concentrations.

The CisBio IP-One HTRF immunoassay quantifies IP_1_, a degradation product of the second messenger d-myo-inositol-1,4,5-trisphosphate, to measure the activation of PLCβ by Gq-coupled GPCRs (25). The IP_1_ assay is a competitive HTRF assay where the d2-labeled IP_1_ analogue acts as the fluorescence acceptor and the terbium cryptate–labeled anti-IP_1_ monoclonal antibody (mAb) acts as the fluorescence donor. The terbium cryptate is a long-lifetime fluorescence donor that can be excited by UV light. LiCl is added during the stimulation period of the assay to block further degradation of IP_1_ by the enzyme inositol monophosphatase. It has been suggested that PLCβ-dependent IP_1_ accumulation also includes contributions from the d-myo-inositol-1,4-bisphosphate formed by direct hydrolysis of phosphatidylinositol-4-phosphate, instead of d-myo-inositol-1,4-bisphosphate formed by the dephosphorylation of d-myo-inositol-1,4,5-trisphosphate formed by hydrolysis of phosphatidylinositol-4,5-bisphophate (10). Because this novel pathway does not depend on phosphatidylinositol-4,5-bisphophate, which is predominantly found at the plasma membrane, the phosphatidylinositol-4-phosphate–dependent IP_1_ accumulation could have a substantial contribution to PLCβ signaling from the endosomal compartment.

#### Agonist dose-response of CysLTR2 DNA titration assay

HEK293T cells were transiently transfected with a serial dilution of 11, 3.6, 1.2, 0.4, and 0.1 ng of WT and mutants of CysLTR2-GFP10 per well as described above. For the DNA titration assay of CysLTR2 WT and L129Q, either WT or L129Q assays were performed in individual plates with mock-transfected cells as controls. For the DNA titration assay of CysLTR2-V2 variants, each experiment was performed in three individual plates, with three sets of two technical replicates each, with WT and mock-transfected cells as controls. Then, 24 h after transfection, the assay plate was placed on an aluminum heating block maintained at 37 °C, and cells were treated with 7 μl/well of various concentrations of LTD4 (final concentrations from 1 μM to 10 pM) diluted in a prewarmed stimulation assay buffer provided by the manufacturer (10-mM HEPES, 1-mM CaCl_2_, 0.5-mM MgCl_2_, 4.2-mM KCl, 146-mM NaCl, 5.5-mM glucose, 50-mM LiCl, pH 7.4) supplemented with 0.2% (w/v) BSA and 50-mM LiCl to prevent IP_1_ degradation. The plate was incubated at 37 °C for 2 h. After incubation, cells were lysed by addition of 3 μl/well of d2-labeled IP_1_ analogue and 3 μl/well of terbium cryptate–labeled anti-IP_1_ mAb diluted in the lysis and detection buffers. The plates were incubated overnight, in the dark, at RT. Time-resolved fluorescence signals were read on the BioTek Synergy NEO plate reader (for cells transfected in DMEM GlutaMAX and assayed in white microplates) or the BioTek Synergy NEO2-TRF Hybrid multi-mode reader (for cells transfected in FluoroBrite DMEM and assayed in black microplates) (BioTek Instruments, Winooski, VT) in the Rockefeller University’s High-Throughput and Spectroscopy Resource Center. The plate is first subjected to flash lamp excitation at 320 nm (BioTek Synergy NEO) or to laser excitation (BioTek Synergy NEO2-TRF) at 337 nm, and then the fluorescence is measured at wavelengths centered at 620 nm and 665 nm simultaneously.

#### Time-course assay

We obtained a time-course of basal and LTD4-dependent IP_1_ accumulation in HEK293T cells transiently transfected with plasmids for CysLTR2 WT, CysLTR2–L129Q, and mock-transfected control. HEK293T cells were transiently transfected with 11 ng of CysLTR2-1D4 (WT and L129Q) per well. Then, 24 h after transfection, the assay plate was placed on an aluminum heating block as described above, and the cells were treated every 20 min over 180 min with 3.5 μl/well of LiCl diluted in a prewarmed stimulation assay buffer at a final concentration of 50 mM. Cells were incubated at 37 °C. At 1 h after the start point, 3.5 μl/well of LTD4 diluted in prewarmed DMEM GlutaMAX at a final concentration of 100 nM (agonist stimulated) or 3.5 μl/well of DMEM GlutaMAX alone (basal) was added in appropriate wells and incubated for 2 h. The reaction was then stopped by successively adding 3 μl/well of the IP_1_ analogue and the anti-IP_1_ mAb in a reverse chronological order.

### BRET^2^ assays

#### General procedure

We then generated a fusion construct of CysLTR2 with GFP10 that can be used in BRET^2^ assays in combination with β-arrestin fused to an engineered variant of *Renilla* luciferase, β-arrestin2-RLuc3 (15). HEK293T cells were transiently cotransfected with β-arrestin2-RLuc3 and CysLTR2-GFP10 WT or the L129Q mutant directly ‘in-plate’ in 96-well plates using Lipofectamine 2000 as described above with slight modifications to account for the larger well volume. The total DNA amount was kept constant at 205 ng per well using an empty vector pcDNA3.1(+). Briefly, a master mix of the β-arrestin2-RLuc3 was made in FluoroBrite DMEM (DMEM without phenol red and suitable for fluorescence experiments) and the CysLTR2–GFP10 DNA were added to these after appropriate distribution. In a separate mixture, the total Lipofectamine 2000 was mixed in FluoroBrite DMEM and incubated for 5 min. The appropriate amount of Lipofectamine 2000/FluoroBrite DMEM mixture was mixed with the DNA/FluoroBrite DMEM and incubated for 20 min. Cells were then trypsinized, resuspended in FluoroBrite DMEM, 20% FBS, 30-mM HEPES, and 8-mM glutamine, and counted. Cells were mixed with the DNA/Lipofectamine 2000/FluoroBrite DMEM mixture and directly plated onto 0.01% poly-d-lysine–coated, black, clear-bottom, tissue culture–treated 96-well plates at a density of 40,000 cells per well in 100-μl FluoroBrite DMEM 10% FBS, 15-mM HEPES, 4-mM glutamine. All assays were conducted 24 h after the transfection. All plasmids were prepared from QIAGEN Plasmid Maxi Kits.

#### Saturation-binding assays

HEK293T cells were transiently transfected with 5 ng of β-arrestin2-RLuc3 and 0, 12.8, 32, 80, or 200 ng of CysLTR2-GFP10 WT/-L129Q, CysLTR2-V2 WT/-L129Q, or CysLTR2-V2(A)_6_ WT/-L129Q per well. Then, 24 h after transfection, media were aspirated carefully from all wells. Then, 30 μl of the prewarmed BRET buffer (DMEM FluoroBrite, 15-mM HEPES, 0.1% (w/v) BSA, 4-mM glutamine) was added to each well. Half of the WT wells were stimulated with LTD4, so 10 μl of LTD4 in the BRET buffer (final concentration 1 μM) was added to these. Then, 10 μl of the BRET buffer was added to all other wells. Cells were incubated for 10 min at RT. After the incubation, BRET^2^ measurements were taken on the BioTek Synergy NEO2 microplate reader using filter set 109 (center wavelength/band width) of 410/80 nm (donor) and 515/30 nm (acceptor). First, the GFP fluorescence was read using the monochromator (ex: 395 nm, em: 510 nm ± 20 nm from the bottom, autogain) to quantify total expression levels. After this procedure, the cell-permeable substrate methoxy e-Coelenterazine (Me-O-e-CTZ/Prolume Purple) was added to each well at a final concentration of 5 μM, and the luminescence at the two wavelengths was read simultaneously.

#### Time-course assays

For the time-course assay, HEK293T cells were transiently transfected with 5 ng of β-arrestin2-RLuc3 and 80 ng of CysLTR2–GFP10 WT, CysLTR2–V2 WT or CysLTR2–V2(A)_6_ WT per well. Then, 24 h after transfection, media were aspirated and 30 μl of the prewarmed BRET buffer was added to each well and the GFP fluorescence was read. Methoxy e-Coelenterazine was added to three columns at 5-μM final concentration followed by addition of 0 nM, 30 nM, and 1000 nM of LTD4 to appropriate wells in the three columns. The plate was quickly placed into the microplate reader so that there was as little lag time between the addition of the ligand and BRET^2^ readings as possible. The three columns take about 60 s to read, and this was repeated 24 times such that a BRET^2^ reading was recorded every 60 s for about 24 min.

#### Agonist dose-response assay

HEK293T cells were transiently transfected with 5 ng of β-arrestin2-RLuc3 and 80 ng of CysLTR2-GFP10 WT/-L129Q, CysLTR2-V2 WT/-L129Q, or CysLTR2-V2(A)_6_ WT/-L129Q per well. Then, 24 h after transfection, media were aspirated and 30 μl of prewarmed BRET buffer was added to each well. Various concentrations of LTD4 (final concentrations from 1 μM to 10 pM) were added to appropriate wells and incubated for 10 min at RT. After the incubation, GFP signals were measured, the substrate was added, and the BRET^2^ signals were obtained.

## Quantification and statistical analysis

### Data reduction, standard calibration, and transformation of HTRF data from IP_1_ assays

The raw signals from the IP_1_ assay were transformed into a fluorescence ratio (665 nm/620 nm), and IP_1_ concentrations were interpolated from a standard curve prepared using the supplied IP_1_ calibrator. The IP_1_ standard curve was fit to a sigmoidal curve using the equation,

(1) $$y\;=\;Bottom\;+\;\frac{\left(Top\;-\;Bottom\right)}{1\;+\;10^{\left(x-logIC_{50}\right)}}$$

The *Bottom* and *Top* parameters are the minimum and maximum fluorescence ratios obtained from the standard curve, respectively, and the IC_50_ and concentration of IP_1_ in nM (*x*) are calculated as logarithmic values.

The fluorescence ratios obtained from individual experiments were then converted into the corresponding IP_1_ concentration (nM, linear) using the following equation and the standard curve:

(2) $$IP1\;=\;\frac{IC_{50}\left(y\;-\;Top\right)}{Bottom\;-\;y}$$

The IC_50_ and IP_1_ concentrations are now calculated as linear values and not logarithmic values. In some cases, these concentrations were further analyzed to obtain normalized IP_1_ values relative to the unstimulated mock-transfected cells (set to 0%) and to the fully stimulated WT receptor (set to 100%).

### Modeling agonist dose-response and receptor density from IP_1_ assays

IP_1_ concentrations or normalized IP_1_ data were fitted to specific models summarized in Figure 2 and introduced below.

### Sigmoidal dose response

For the LTD4 dose response of HEK293T cells transfected with varying amounts of CysLTR2 encoding plasmid DNA, the IP_1_ concentrations in nM were plotted against the logarithmic concentration of LTD4. These data were first fit to a three-parameter sigmoidal dose-response function described below:

(3) $$y\;=\;Bottom\;+\;\frac{\left(Top\;-\;Bottom\right)}{1\;+\;10^{\left(logEC_{50}-x\right)}}$$

The *Bottom* and *Top* parameters describe the lower and upper asymptotic values, respectively. The logarithmic form of the *log EC*_*50*_ parameter ensures positive solution for the *EC*_*50*_. Moreover, the logarithmic fitting parameters account for the fact that the solutions for *EC*_*50*_ should be log-normally distributed. We use an alternative form for dose-response experiments with competitors or inverse agonists, where the *log IC*_*50*_ parameter replaces *log EC*_*50*_ and ensures positive fitting solutions for the half maximal inhibitory concentration *IC*_*50*_.

(4) $$y\;=\;Bottom\;+\;\frac{\left(Top\;-\;Bottom\right)}{1\;+\;10^{\left(x-logIC_{50}\right)}}$$

To account for data sets that show no significant dose response, we also fit each data set with a horizontal line function as an alternative hypothesis. We chose the best model, either sigmoidal curve or horizontal line, by the Akaike Information Criterion (Table S1*A*). Note that the horizontal line fitting function in the GraphPad Prism 8 software has the form y=Mean+0(x) because the software requires the use of the independent variable *x*, which is multiplied by zero to negate its influence. Effectively, this fit is a horizontal line plotting the mean IP_1_ concentrations for all LTD4 doses.

### Slack–Hall operational model

Figure 2 introduces two operational models: the Black–Leff and Slack–Hall models. The key insight of Zhou *et al.* is that the Slack–Hall model can be used to quantify the agonist-independent, inherent pathway bias of the constitutive signaling of a receptor referred to as *receptor bias* (12, 13). The Slack–Hall model is an expansion of the classical Black–Leff operational model, which underlies methods to calculate functional selectivity or agonist bias (17). In the Slack–Hall model, both the free receptor [R] and agonist-bound receptor [AR] can produce a stimulus, S=ε[AR]+[R]. The parameter *ε* describes the efficacy of an agonist (A) to produce a stimulus. The Slack–Hall model splits the τ parameter of the Black–Leff model into a product of two parameters, χ and ε. The basal response is determined by χ and is defined as the ratio of [R]_*t*_, the total receptor concentration, and *K*_*e*_, the receptor concentration producing half-maximal effect in the *absence* of an agonist. In contrast, the τ parameter in the Black–Leff model is the ratio of [R]_*t*_ and a different *K*_*e*_, which is defined as the receptor concentration producing half-maximal effect in the *presence* of a saturating agonist concentration. The ε parameter measures the *intrinsic efficacy* of the ligand. We slightly modified the original form of this equation to account for fitting problems for χ, the basal response parameter, in cases where the CA is very low. Taking the *log τ* parameter from the Black–Leff model, we implicitly calculate *log χ* from *log τ*–*log ε*. The final equation of our modified Slack–Hall model is as follows:

(5) $$y\;=\;Basal\;+\;\frac{E_{max}{\left(10^{log\tau -log\varepsilon }\;+\;10^{log\tau +x+logK_{A}}\right)}^{n}}{{\left(10^{log\tau -log\varepsilon }\;+\;10^{log\tau +x+logK_{A}}\right)}^{n}\;+\;{\left(1\;+\;10^{x+logK_{A}}\right)}^{n}}$$

Table S1B shows the parameters for the Slack–Hall operational model fitted to the experiments shown in Fig. 1, *A*–*B*, 6, *A–D*. In these fits, *x* is the log of the agonist concentration and *y* is the response to the agonist. *E*_*max*_, maximal IP_1_ concentration for the system, was first fit individually for each condition, and then the highest *E*_*max*_ value from this was used as a shared, fixed value for all final fits. The parameter *log K*_*A*_ is the logarithm of the agonist-receptor association constant, *K*_*A*_. Note that *K*_*A*_ is the inverse of the dissociation constant *K*_*a*_. The fitting parameters *log ε* and *log K*_*A*_ were shared for all conditions, whereas *log τ* was left free to give an independent value for each condition. The optimal value and error for *log χ* was separately calculated from the difference *log τ*–*log ε*.

### Modeling the time course of IP_1_ accumulation using IP_1_ assays

For the IP_1_ accumulation time course (Fig. S1, A–B), the corrected IP_1_ concentrations, in nM, were plotted against time in minutes. These data are then fitted to a one-phase decay model using the following equation:

(6) $$y\;=\;\left(y_{0}-Plateau\right)e^{-kx}\;+\;Plateau.$$

Here *y*_0_ is the IP_1_ concentration at time zero, while *Plateau* is the IP_1_ concentration at infinite time, *k* is the rate constant of the decay, *x* is the time of incubation, and *y* is the IP_1_ concentration.

### Data reduction for BRET^2^ assays

Raw BRET^2^ ratios were determined by calculating the ratio of the light intensity emitted by the GFP10 (515 nm) over the light intensity emitted by the RLuc3 (410 nm). The BRET^2^ signals minus the basal BRET^2^ signals (β-arrestin-RLuc3 only signals) give the net BRET^2^ values.

### Two-phase decay model for time course

For all time-course assays, the BRET^2^ ratios (*y*) were plotted against time (*x*), in seconds, to assess the time dependence of the LTD4-stimulated β-arrestin recruitment (Figs. 1*C* and 6, *G*–H). These data were fitted to a two-phase decay model using the following equation:

(7) $$y=\left\{ \begin{array}{l} \left(-e^{-\left(10^{logk_{fast}}\right)\left(x-x_{0}\right)}\;+\;e^{-\left(10^{logk_{slow}}\right)\left(x-x_{0}\right)}\right)\\ \frac{Plateau\cdot 10^{logk_{fast}}}{10^{logK_{fast}}\;-\;10^{logk_{slow}}}\;+\;y_{0},\;for\;x>x_{0}\\ y_{0}\;otherwise \end{array}\right.$$

This model is the sum of two decay processes, one fast and the other slow. The fast process describes the rise of the curve, whereas the slow process determines the subsequent decay. We use logarithmic fitting parameters, *log k*_*fast*_ and *log k*_*slow*_, to constrain the fitting space to positive values of the rate constants, *k*_*fast*_ and *k*_*slow*_, which are rate constants for the two decay processes. The *Plateau* parameter scales the peak height, and *y*_*0*_ is the β-arrestin recruitment at time zero. *k*_*fast*_ describes the initial recruitment of β-arrestin, which is dependent on the concentration of the active receptor and is represented as the initial increase in the signal. *k*_*slow*_ describes the disassembly of the receptor–β-arrestin complex and is represented by the decay of the signal over time. For this fit, *x*_*0*_ and *log k*_*slow*_ are shared for all three curves (0-nM, 30-nM, and 1000-nM LTD4). The *y*_*0*_ is determined by first fitting the 0-nM LTD4 curve to a horizontal line and then using this constant as the fixed *y*_*0*_ value for the fits of the 30-nM and 1000-nM LTD4 data where *log k*_*fast*_ is varied independently for each condition. The data for the fits are provided in Table S1*C*.

### Sigmoidal dose-response and normalization

For the agonist dose-response assays, the BRET^2^ ratios were plotted against logarithmic concentrations of LTD4 (Figs. 1*D* and 6, *E*–F). The data were fit to a sigmoidal curve (Equation 3), with an alternative model as a horizontal line, y=Mean+0(x), as described above. Table S1*A* summarizes the fitting parameters.

### CA and receptor bias for Gq and β-arrestins

We quantified the CA using a modified Slack–Hall operational model to enable the calculation of the *receptor bias* between Gq/11 and β-arrestin pathways for L129Q relative to WT and to characterize the effect of the V2 tail variants. The term *receptor bias* was introduced to describe the pathway preference of the basal signaling activity of a receptor (12), in contrast to the *agonist bias* that describes ligand-dependent pathway preferences of a receptor (17). The parameter χ determines the value of the basal response. The challenge is that χ is proportional to the receptor density, which requires standardization for the comparison of receptor mutants with potential impact on receptor expression levels. In our experiments, we control the receptor density by the gene dosage and measure the fluorescence from the GFP10 fusion to calibrate the relative expression levels (Fig. 3). The GFP10 readings were first normalized by dividing the sample F(GFP10) by the basal F(GFP10) (β-arrestin2-RLuc3 only), and then further divided this by the normalized F(GFP10) of the exchange protein activated by cAMP (EPAC) BRET^2^ biosensor (RLuc3-EPAC-GFP10 (15) developed from the guanine nucleotide EPAC that acts as the positive control) to give the following:

(8) $${F(GFP10)}_{norm}\;=\;\frac{\frac{{F(GFP10)}_{sample}}{{F(GFP10)}_{RLuc3}}\;-\;1}{\frac{{F(GFP10)}_{EPAC}}{{F(GFP10)}_{RLuc3}}\;-\;1}$$

The inner averages are for technical replicates per experiment, and the outer averages are for all experiments. These data are fit to a sigmoidal model, where F(GFP10) is the response as a function of DNA dosage. The log EC_50_ parameter is shared in the global fit, the bottom parameter is set to zero, whereas the top parameter is left unconstrained to capture the different expression levels of each variant. We use these sigmoidal fits to interpolate F(GFP10) for lower DNA/cell levels used in the Gq second messenger IP_1_ assays, and thus the interpolated F(GFP10) were plotted against *log τ* values and *log χ* values from fitting the data to the modified Slack–Hall model (Fig. 4, Table S1*B*). Assuming the F(GFP10) is proportional to the total receptor concentration by some scaling constant, *c*, and rearranging $\chi =\frac{{\left[R\right]}_{T}}{K_{e}}$ gives the following:

(9) $$log\chi \;=\;logc\;+\;\log\left(F(GFP10)\right)\;-\;logK_{e}$$

Thus, plotting *log χ* against *log (F(GFP10))* and fitting to a line with a slope of 1 gives y-intercept of *log c* – *log K*_e_. We can similarly plot *log τ* against *log (F(GFP10))* to get a y-intercept of *log c* – *log K*_e_ + *log ε*. These allow for an accurate quantification of *log ε;* and of differences of *log K*_e_ for different receptor constructs at a standard density.

We noticed that in the absence of a ligand, the Slack–Hall model reduces to the mathematical form of a one-site saturation-binding function (18). We then plotted the BRET^2^ ratios against normalized F(GFP10) readings of each CysLTR2 variant (Fig. 5). These data are fitted to a one-site saturation-binding isotherm using the following equation:

(10) $$y\;=\;\frac{10^{logB_{max}}}{x\;+\;10^{logK_{d}}}x\;+\;background$$

The logarithmic fitting parameters, *log B*_*max*_ and *log K*_*d*_, ensure positive fitting solutions for *B*_*max*_ and *K*_*d*_*. B*_*max*_ is the maximal increase of the BRET^2^ ratio due to β-arrestin binding. *log B*_*max*_ is a shared value for the global fits of all variants. *K*_*d*_ is the equilibrium dissociation of the F(GFP10), which gives half-maximal β-arrestin binding. The *log K*_*d*_ varied independently for each curve. The parameter *background* is constrained to 0.0068, the BRET^2^ ratio for the β-arrestin2-RLuc3 sample without receptor.

Tight independent estimates of *K*_*d*_ and *B*_*max*_ are not required because at low concentrations, only the ratio *B*_*max*_:*K*_*d*_ determines the concentration-dependent binding, which can be estimated from the initial slope. The initial slopes are well defined by samples even at low expression levels of receptors and avoid the need for very high receptor concentrations to reach saturation. The initial slope of a saturation binding experiment as a function of the total receptor concentration is *B*_*max*_*/K*_*d*_. The initial slope of the Slack–Hall model as function of the total receptor concentration is *E*_*max*_*/K*_*e*_. The equivalence of *B*_*max*_*/K*_*d*_ and *E*_*max*_*/K*_*e*_ enables subsequent calculations of Δlog χ, the differences of log χ for the WT and mutant receptors that determine changes in the CA, and the double difference ΔΔlog χ that determines the receptor bias. Those differences eliminate the constant *B*_*max*_ and *E*_*max*_ terms and focus on changes of the bias-relevant terms *K*_*d*_ and *K*_*e*_.

Using Figures 4 and 5, we are able to compare the CAs for the different receptor constructs at a standard density. We further normalize the CAs relative to the fully agonist-stimulated WT receptor. These values are reported above the corresponding graphs in Figures 1, *A–B* and *D* and 6, *A–F*.

## Data availability

All data needed to evaluate the conclusions in the article are presented in the article or the Supporting Information. Additional requests should be made to the corresponding author.

## Conflict of interest

The authors declare that they have no conflicts of interest with the contents of this article.
