# Supplementary material for: Direct evidence that the GPCR CysLTR2 mutant causative of uveal melanoma is constitutively active with highly biased signaling
Source: J Biol Chem. 2020 Dec 11;296:100163. doi: 10.1074/jbc.RA120.015352 (PMC7948404; doi:10.1074/jbc.RA120.015352)
Supplement: Figures and Tables [file mmc1.pdf]

SUPPORTING INFORMATION

Direct evidence that the GPCR CysLTR2 mutant causative of uveal melanoma is constitutively active with highly biased signaling

Emilie Ceraudo[a,1], Mizuho Horioka[a,b,1], Jordan M. Mattheisen[a,b],
Tyler D. Hitchman[c,d], Amanda R. Moore[c,e,f], Manija A. Kazmi[a],
Ping Chi[c,d,e,g,h], Yu Chen[c,d,e,g,h], Thomas P. Sakmar[a,i*], and Thomas Huber[a*].

[a]Laboratory of Chemical Biology and Signal Transduction, The Rockefeller University, New York, NY 10065, USA.
[b]Tri-Institutional PhD Program in Chemical Biology, New York, NY 10065, USA.
[c]Human Oncology and Pathogenesis Program, Memorial Sloan Kettering Cancer Center, New York, NY 10065, USA.
[d]Louis V. Gerstner Jr. Graduate School of Biomedical Sciences, Memorial Sloan Kettering Cancer Center, New York, NY 10065, USA.
[e]Weill Cornell Graduate School of Medical Sciences, Cornell University, New York, NY 10065, USA.
[f]Current address: Department of Discovery Oncology, Genentech Inc, South San Francisco, CA 94080, USA.
[g]Department of Medicine, Memorial Sloan Kettering Cancer Center, New York, NY 10065, USA.
[h]Department of Medicine, Weill Cornell Medical College, 1300 York Avenue, New York, NY 10065, USA.
[i]Division of Neurogeriatrics, Department of Neurobiology, Care Sciences and Society, Karolinska Institutet, 171 64 Solna, Sweden.
[1]These authors contributed equally
[*]Correspondence: sakmar@rockefeller.edu (T.P.S.) and hubert@rockefeller.edu (T.H.)

Supplemental Information:
Fig. S1-S3
Table S1, S2

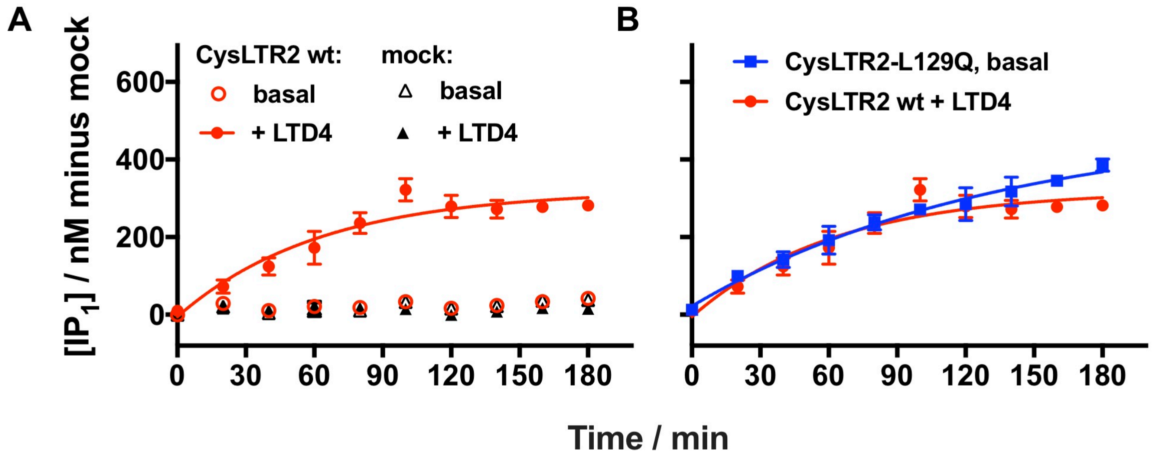

Fig. S1. Development of the CysLTR2 functional assay for Gq signaling pathway. (**A**,**B**) Lithium chloride-dependent accumulation of IP_1 differs for agonist-induced and constitutive receptor activity. Lithium chloride (LiCl) is added during the stimulation period of the assay to block further degradation of IP_1. Time-course of the effect of 50 mM LiCl on the basal and LTD4-induced IP_1 accumulation for CysLTR2 WT (**A**) and -L129Q (**B**) transfected HEK293T cells over 180 min. (**A**) Basal IP_1 accumulation of CysLTR2 WT (open red circles) is comparable to mock-transfected cells with (solid black triangles) or without (open black triangles) LTD4 stimulation and is not affected by the addition of LiCl. CysLTR2 WT stimulated by LTD4 exhibits an increasing IP_1 accumulation over 100 min after addition of LiCl, before reaching a plateau (solid red circles). (**B**) CysLTR2-L129Q (blue squares) shows a LTD4-independent IP_1 accumulation similar to the WT receptor that continues to increase over 180 min. The data are expressed as the mean ± SEM of IP_1 (nM) minus mock and result from one experiment performed in four technical replicates.

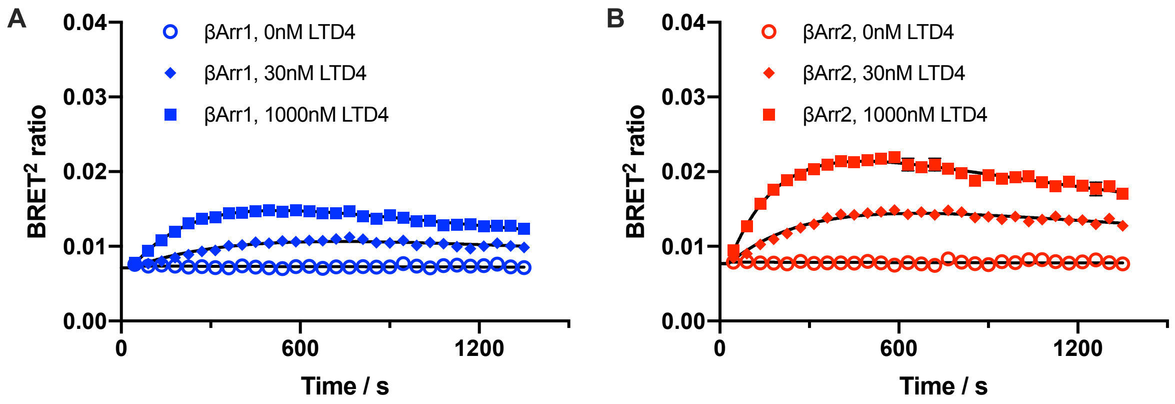

Fig. S2. Time-course of LTD4-stimulated β-arrestin-recruitment measured by the BRET^2 assay with CysLTR2-GFP10 and β-arrestin-RLuc3. (**A**) The time-dependent increase of net BRET^2 demonstrates recruitment of β-arrestin1 (βArr1) and was measured for two LTD4 concentrations (30 nM, blue diamond; 1000 nM, blue square) and vehicle (0 nM, blue open circle). (**B**) Time-course of β-arrestin2 (βArr2) recruitment for two LTD4 concentrations (30 nM, red diamond; 1000 nM, red square) and vehicle (0 nM, red open circle). The time-dependent data in (**A**,**B**) were globally fitted with a double exponential function using shared slow kinetic rates and starting values. They are the mean ± SEM from two independent experiments, with two sets of four technical replicates.

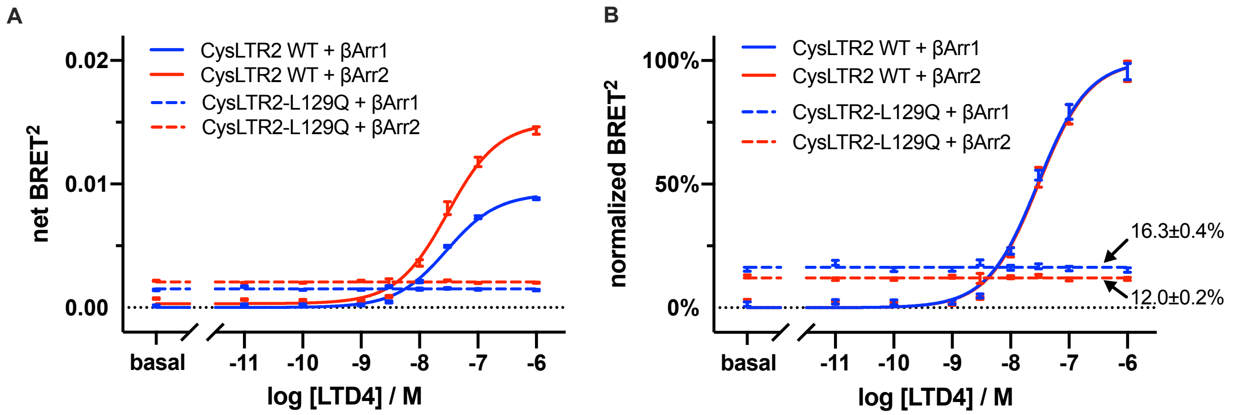

Fig. S3. β-arrestin-recruitment BRET^2 assay with CysLTR2-GFP10 and β-arrestin-RLuc3. (**A**) The LTD4 dose-dependent increase of net BRET^2 demonstrates the recruitment of β-arrestin1 (βArr1, solid red line and points; EC_{50} is 29 nM (95% C.I. 25 to 34 nM)) and β-arrestin2 (βArr2, solid blue line and points; EC_{50} is 30 nM (95% C.I. 25 to 36 nM)) to CysLTR2 WT. In comparison, the data for CysLTR2-L129Q indicate ligand-independent recruitment of β-arrestin1 (blue dashed line and open points) and β-arrestin2 (red dashed line and open points) to CysLTR2-L129Q. (**B**) The BRET^2 data were independently normalized for either β-arrestin1 or 2 using the asymptotic endpoints of the sigmoidal fits for the WT receptor. The normalized data show a nearly perfect overlap of the fitted curves for both β-arrestins binding the WT receptor. The ligand-independent β-arrestin recruitment for CysLTR2-L129Q is 16.3 ± 0.4% and 12.0 ± 0.2% for β-arrestin1 and 2, respectively. The dose-response data are the mean ± SEM from three independent experiments, with nine concentrations and three technical replicates each.

Table S1. Parameters obtained from fitting experimental data to pharmacological models. The different models introduced in **Figure 2** were used to fit the experimental data shown in **Fig. 1 and 6**. (**A**) Parameters for sigmoidal *versus* horizontal line model used in LTD4 dose-response experiments. The horizontal line accounts for data that show no significant dose-response and are represented by Mean, only. The Akaike Information Criterion were used to determine which model fit the data set best (**bold**). (**B**) Parameters for the modified Slack-Hall model. Shared parameters are indicated in respective rows, and E_{max} is fixed for all data sets. The global, fixed E_{max} was determined by first fitting all CysLTR2-GFP10 dose-responses individually to find the largest E_{max} (*). ε and its C.I.s were later calculated from *log* ε fit parameters. (**C**) Parameters for two-phase decay model for the CysLTR2-GFP10 $BRET^2$ time-courses. The time-course for the unstimulated (0 nM) cells were fit to a horizontal line, to determine y_0. This y_0 was used as a fixed value when fitting the stimulated (30, 1000 nM) cells to the two-phase decay model.

Table S1A. Sigmoidal *versus* Horizontal Line Model

	Bottom	Top	log EC_{50}	Amplitude	Mean	Dof [a)]
CysLTR2-GFP10 $BRET^2$ Dose-Response (**Fig. 1D**)						
WT	**0.000679 ± 0.000227**	**0.0186 ± 0.0004**	**-7.708 ± 0.043**	**0.0179 ± 0.0004**	0.00698 ± 0.00059	143
L129Q	0.00228 ± 0.0000664	0.00243 ± 0.00019	-7.015 ± 1.978	0.000144 ± 0.000188	**0.00231 ± 0.00005**	95
CysLTR2-V2(A)$_6$-GFP10 $BRET^2$ Dose-Response (**Fig. 6E**)						
WT	**0.00165 ± 0.00051**	**0.0143 ± 0.0012**	**-7.305 ± 0.151**	**0.0126 ± 0.0012**	0.00510 ± 0.00057	95
L129Q	0.00391 ± 0.00009	N.C.	N.C.	N.C.	**0.00387 ± 0.00007**	63
CysLTR2-V2-GFP10 $BRET^2$ Dose-Response (**Fig. 6F**)						
WT	**0.00874 ± 0.00061**	**0.0374 ± 0.0009**	**-8.031 ± 0.0743**	**0.0286 ± 0.0011**	0.0205 ± 0.0013	95
L129Q	0.0184 ± 0.0002	0.0195 ± 0.0007	-6.894 ± 0.964	0.00106 ± 0.00068	**0.0187 ± 0.0002**	69

Bold = Indicates the parameters of the preferred model (either sigmoidal dose-response or horizontal line). Normal = Parameters of the rejected model.
a) Degrees of Freedom, N.C. = Non-converging

Table S1B. Slack-Hall Operational Model

	log K_A	log χ	log ε	Basal	E_{max}	log τ	Dof [a)]	ε
CysLTR2-GFP10 WT (**Fig. 1A**)								
11ng		-2.098 ± 0.182				-0.5979 ± 0.01157		
3.6ng		-2.291 ± 0.185				-0.7904 ± 0.01511		
1.21ng		-2.474 ± 0.188				-0.9739 ± 0.02083		
0.4ng		-2.776 ± 0.199				-1.275 ± 0.03822		
0.1ng		-3.106 ± 0.226				-1.605 ± 0.07844		
Global (fixed)					= 1531			
Global (shared)	7.607 ± 0.038		1.501 ± 0.178	33.22 ± 2.88			232	31.70 (95% C.I. 14.19–70.78)
CysLTR2-GFP10-L129Q (**Fig. 1B**)								
11ng		-0.658 ± 0.0148				-0.6129 ± 0.0131		
3.6ng		-0.7802 ± 0.0162				-0.7351 ± 0.0146		
1.21ng		-0.9665 ± 0.0197				-0.9214 ± 0.0184		
0.4ng		-1.274 ± 0.032				-1.229 ± 0.031		
0.1ng		-1.656 ± 0.068				-1.610 ± 0.067		
Global (fixed)				= 33.22	= 1531			
Global (shared)	8.955 ± 0.774		0.04507 ± 0.01596				233	1.109 (95% C.I. 1.032–1.192)
CysLTR2-V2(A)$_6$-GFP10 WT (**Fig. 6A**)								
11ng		-3.257 ± 0.637				-0.7007 ± 0.0608		
3.6ng		-2.914 ± 0.627				-0.3576 ± 0.0655		
1.21ng		-2.635 ± 0.620				-0.07933 ± 0.07907		
0.4ng		-2.355 ± 0.612				0.2011 ± 0.1027		
0.1ng		-2.182 ± 0.606				0.3744 ± 0.1226		
Global (shared)	7.596 ± 0.069		2.556 ± 0.642	-2.169 ± 7.923	1531 ± 142*		411	359.7 (95% C.I. 19.8–6521.1)
CysLTR2-V2(A)$_6$-GFP10-L129Q (**Fig. 6C**)								
11ng		-0.8145 ± 0.0350				-0.7290 ± 0.0358		
3.6ng		-0.4482 ± 0.0228				-0.3627 ± 0.0244		
1.21ng		-0.2283 ± 0.0200				-0.1428 ± 0.0221		
0.4ng		-0.02402 ± 0.01916				0.0615 ± 0.0216		
0.1ng		-0.03373 ± 0.01917				0.05179 ± 0.02163		

Global (fixed)				= 13.44	= 1531			
Global (shared)	8.402 ± 0.562		0.08552 ± 0.01980				410	1.218 (95% C.I. 1.114–1.331)
CysLTR2-V2-GFP10 WT (**Fig. 6B**)								
11ng		-3.538 ± 0.305				-1.747 ± 0.113		
3.6ng		-3.129 ± 0.264				-1.338 ± 0.046		
1.21ng		-2.736 ± 0.251				-0.9453 ± 0.0210		
0.4ng		-2.382 ± 0.247				-0.591 ± 0.012		
0.1ng		-2.163 ± 0.246				-0.3724 ± 0.0092		
Global (fixed)					= 1531			
Global (shared)	8.121 ± 0.032		1.791 ± 0.243	1.355 ± 3.069			411	61.80 (95% C.I. 20.64–185.0)
CysLTR2-V2-GFP10-L129Q (**Fig. 6D**)								
11ng		-2.119 ± 0.416				-1.855 ± 0.405		
3.6ng		-1.460 ± 0.105				-1.196 ± 0.093		
1.21ng		-1.024 ± 0.050				-0.7589 ± 0.0384		
0.4ng		-0.7854 ± 0.0366				-0.5208 ± 0.0253		
0.1ng		-0.6355 ± 0.0314				-0.3709 ± 0.0203		
Global (fixed)					= 1531			
Global (shared)	8.332 ± 0.109		0.2647 ± 0.0171	13.44 ± 14.51			411	1.840 (95% C.I. 1.703–1.987)

a) Degrees of Freedom, N.C. = Non-converging

* The data for all CysLTR2-GFP10 dose-responses were first fit individually to determine the largest E_{max}, indicated here with the asterisk. All fits to the Slack-Hall model shown in this table share this E_{max} value as a global, fixed value.

Table S1C. Two-Phase Decay Model for Time-courses

	y_0	x_0	Plateau	log K_{fast}	log K_{slow}	Dof [a)]	K_{fast}	K_{slow}
CysLTR2-GFP10 BRET^2 time-course (**Fig. 1C**)								
0 nM*	0.00842 ± 0.00004					551		
30 nM			0.0101 ± 0.0003	-2.373 ± 0.037		1098	0.00424 (95% C.I. 0.00359-0.00501)	
1000 nM			0.0180 ± 0.0004	-1.992 ± 0.042		1098	0.0102 (95% C.I. 0.0084-0.0123)	
Global (fixed)	= 0.00842							
Global (shared)		75.78 ± 5.86			-3.821 ± 0.074			0.000151 (95% C.I. 0.000108-0.000211)
CysLTR2-V2(A)$_6$-GFP10 BRET^2 time-course (**Fig. 6G**)								
0 nM*	0.00880 ± 0.00004					551		
30 nM			0.00663 ± 0.00027	-2.590 ± 0.035		1098	0.00257 (95% C.I. 0.00220-0.003003)	
1000 nM			0.0118 ± 0.0004	-2.318 ± 0.031		1098	0.00481 (95% C.I. 0.0042-0.0055)	
Global (fixed)	= 0.00880							
Global (shared)		37.90 ± 4.30			-3.957 ± 0.137			1.00 (95% C.I. 0.54-1.85)
CysLTR2-V2-GFP10 BRET^2 time-course (**Fig. 6H**)								
0 nM*	0.0148 ± 0.0001					551		
30 nM			0.0237 ± 0.0003	-2.471 ± 0.019		1098	0.00338 (95% C.I. 0.00311-0.00368)	
1000 nM			0.0276 ± 0.0002	-1.996 ± 0.0243		1098	0.0101 (95% C.I. 0.0090-0.0113)	
Global (fixed)	= 0.0148							
Global (shared)		32.58 ± 3.45			$-\infty$			0

a) Degrees of Freedom

* These data are fit to a horizontal line, not the two-phase decay model, so the other parameters listed are not applicable.

Table S2. All oligonucleotides used to generate the various constructs used in this paper. (**A**) TagMaster site-directed mutagenesis primers used in deleting FLAG epitope tag from FLAG-CysLTR2-1D4. (**B**) NEBuilder HiFi DNA Assembly primers used to amplify fragments and build CysLTR2-GFP10. (**C**) Top: Gene optimized 27 amino acid sequence from C-terminal tail of vasopressin V2 receptor and its hexa-Ala version. Bottom: NEBuilder HiFi DNA Assembly primers used to generate CysLTR2-V2-GFP10 and CysLTR2-V2(A)$_6$-GFP10. All oligonucleotides were ordered from IDT at the standard desalting grade.

Table S2A. Primers for deletion of FLAG epitope tag from FLAG-CysLTR2-1D4

Purpose of Primer	Oligonucleotide Sequence of Primers
FLAG-deletion-Fwd	5' TCT GCA GAT ATC GCC ACC ATG GAG AGG AAG TTC ATG TCC CTG 3'
FLAG-deletion-Rev	3' CAG GGA CAT GAA CTT CCT CTC CAT GGT GGC GAT ATC TGC AGA G 5'

Table S2B. NEBuilder HiFi DNA Assembly primers used in building BRET^2 acceptors (CysLTR2-GFP10)

Name	Sequence of Primers (5’ to 3’) w/ overlaps underlined	Purpose
TH1700_1	GGT GGC GGC GGT ATC ATC TCG TGC AGG GCG GCC GCT AAG CTT AAG TTT AAA CGC TAG CCA GC	Anneals to FLAG-CysLTR2-1D4 and overlaps with HA-CLIP-CLR
TH1700_2	TAG CGT TTA AAC TTA AGC TTA GCG GCC GCC CTG CAC GAG ATG ATA CCG CCG CCA CCA TGT CCC TGC AGC CCA GC	Anneals to HA-CLIP-CLR and overlaps with FLAG-CysLTR2-1D4
TH1700_3	CGA ATT CAC CGG TAC CCA CCC TTG TCT CTT TTC TGA GC	Anneals to CXCR4-GFP10, and overlaps with FLAG-CysLTR2-1D4
TH1700_4	GAC AAG GGT GGG TAC CGG TGA ATT CGT GAG CAA GGG CGA GGA G	Anneals to FLAG-CysLTR2-1D4, and overlaps with CXCR4-GFP10
TH1700_5	ACG GTG GTG CTG GCC TCA TCG GAT CCG CCT GCA GGC TTG TAC AGC TCG TCC ATG C	Anneals to HA-CLIP-CLR, and overlaps with CXCR4-GFP10
TH1700_6	CCT GCA GGC GGA TCC GAT GAG GCC AGC ACC ACC	Anneals to CXCR4-GFP10, and overlaps with HA-CLIP-CLR
1740_FLAG-CysLTR2_fwd	ATG ATA CCG CCG CCA CCA TGG AGA GGA AGT TCA TGT CC	Anneals to FLAG-CysLTR2-1D4, and overlaps with TH1705 (CLTR2-GFP10-1D4)
1740_FLAG-CysLTR2_rev	CTC ACG AAT TCA CCG GTA CCC ACC CTT GTC TCT TTT CTG	Anneals to FLAG-CysLTR2-1D4, and overlaps with TH1705 (CLTR2-GFP10-1D4)
1720_TH1705_fwd	GGT ACC GGT GAA TTC GTG AG	Anneals to TH1705 (CLTR2-GFP10-1D4)
1720_TH1705_rev	CAT GGT GGC GGC GGT ATC	Anneals to TH1705 (CLTR2-GFP10-1D4)

Table S2C. NEBuilder HiFi DNA Assembly primers and oligos used for CysLTR2-V2-GFP10 and CysLTR2-V2$(A)_6$-GFP10

Name	Sequence of Oligonucleotides	Purpose
V2 Tail	GGC AGA ACA CCT CCA TCT CTG GGA CCT CAG GAT GAG AGC TGT ACC ACA GCC TCT AGC AGC CTG GCC AAG GAT ACA AGC TCT	Gene optimized 27 amino acid sequence from C-terminal tail of vasopressin V2 receptor
V2$(A)_6$ Tail	GGC AGA ACA CCT CCA TCT CTG GGA CCT CAG GAT GAG AGC TGT ACC ACA GCT GCT GCC GCT CTG GCC AAA GAT GCT GCT GCT	Gene optimized, phosphorylation-resistant $(A)_6$ 27 amino acid sequence from C-terminal tail of vasopressin V2 receptor
**Name**	**Sequence of Primers (5' to 3') w/ overlaps underlined**	**Purpose**
CysLTR2(1-346)V2-GFP10-1D4_fwd	TGG CCA AGG ATA CAA GCT CTG GTA CCG GTG AAT TCG TG	Anneals to CysLTR2-GFP10 after the full length CysLTR2, and overlaps with V2 tail
CysLTR2(1-346)V2-GFP10-1D4_rev	AGA GAT GGA GGT GTT CTG CCC ACC CTT GTC TCT TTT CTG	Anneals to CysLTR2-GFP10 after the full length CysLTR2, and overlaps with V2 tail
CysLTR2(1-346)V2$(A)_6$-GFP10-1D4_fwd	TGG CCA AAG ATG CTG CTG CTG GTA CCG GTG AAT TCG TG	Anneals to CysLTR2-GFP10 after the full length CysLTR2, and overlaps with V2$(A)_6$ tail
CysLTR2(1-346) V2$(A)_6$-GFP10-1D4_rev	AGA GAT GGA GGT GTT CTG CCC ACC CTT GTC TCT TTT CTG	Anneals to CysLTR2-GFP10 after the full length CysLTR2, and overlaps with V2$(A)_6$ tail
